# Risk Factors and Outcomes of Postoperative Catheter-Associated Urinary Tract Infection in Colorectal Surgery Patients: A Retrospective Cohort Study

**DOI:** 10.7759/cureus.15111

**Published:** 2021-05-19

**Authors:** Ali H Farsi

**Affiliations:** 1 Department of Surgery, Faculty of Medicine, King Abdulaziz University, Jeddah, SAU

**Keywords:** urinary tract infection, colorectal surgery, ureteric catheterization, urinary catheter, surgical outcomes

## Abstract

Introduction

Catheter-associated urinary tract infection (CAUTI) is a relatively common cause of postoperative morbidity in colorectal surgery patients. It has been associated with increased length of stay and mortality.

Methods

We performed a retrospective cohort study of 620 colorectal surgeries to assess the prevalence of CAUTI and its relationship with preoperative and operative factors. We also sought to identify its association with postoperative outcomes.

Results

We found that CAUTI occurred in 20.6% of colorectal procedures. We found that CAUTI was associated with older patient age, female gender, higher BMI, higher American Society of Anesthesiologists (ASA) classification, lower hemoglobin, higher creatinine, lower albumin, urgent procedures, bilateral ureteric stent placement, usage of double-J (DJ) stents, postoperative abdominal sepsis, and perioperative steroid usage. CAUTI was also associated with the presence of underlying medical conditions such as hypertension, ischemic heart disease, chronic kidney disease, cerebrovascular disease, and diabetes. With regards to postoperative outcomes, it was associated with postoperative stroke, myocardial infarction, prolonged length of stay, Intensive care unit stay, and mortality.

Conclusion

CAUTI remains a significant cause of morbidity in colorectal patients. Our patient population had a significantly higher risk of CAUTI compared to other series. Though sometimes labelled a minor postoperative complication, its occurrence is associated with other more significant postoperative complications, including death.

## Introduction

Catheter-associated urinary tract infections (CAUTI) are one of the most common hospital-acquired infections [1], with colorectal surgery patients being at particular risk [1, 2]. They are often considered minor complications but remain a significant cause of morbidity as they are associated with an increased postoperative length of stay (PLOS) and increased hospital cost. CAUTI is also associated with an increased risk of mortality [2-4]. They are also believed to contribute to other morbidities, such as venous thromboembolism, pneumonia, and delirium in older medical patients by limiting mobility [5].

Colorectal surgery patients often require urinary tract instrumentation in order to place ureteric catheters to guard against ureteric injury during surgery, or Foley catheters to enable urine output measurement intraoperatively and postoperatively and to avoid postoperative urinary retention [6].

In this study we aimed to identify the rates and frequency of CAUTI among colorectal surgery patients and the association of CAUTI with other adverse postoperative events.

## Materials and methods

Setting

We obtained ethical approval from the Unit of Biomedical Ethics Research Committee at the Faculty of Medicine, King Abdulaziz University (reference no 263-21). We performed a retrospective cohort study by reviewing the operating room list from January 2013 to January 2020 at King Abdulaziz University Hospital, an academic tertiary care center, to identify all colorectal surgeries.

Inclusion and exclusion criteria

Our inclusion criteria were adult patients over the age of 18 undergoing surgery on the colon and rectum. Our exclusion criteria were patient whose surgery did not require entry into the peritoneal cavity such as stomal revision or transanal excision and patients who did not have a Foley catheter placed. If a patient had more than one procedure, we excluded the second procedure if it occurred within six months of the first procedure. We did this in order to eliminate any surgeries related to complications from index procedure such as an operation to treat an anastomotic leak, evisceration, early stoma complications, and so forth.

Data collection and outcomes of interest

We reviewed the patients’ charts and extracted baseline characteristics including age, gender, body mass index (BMI), American Society of Anesthesiologists (ASA) classification, medical history, including hypertension, diabetes, ischemic heart disease (IHD), heart failure, chronic liver disease (CLD), chronic kidney disease (CKD), and asthma. We extracted the patients’ preoperative laboratory values, including hemoglobin, white blood cell count (WBC), platelets, international normalized ratio(INR), activated partial thromboplastin time (aPTT), albumin and creatinine. We reviewed the operative note to extract operative details such as procedure urgency, duration, procedure type, method of surgery and the use of ureteric catheters. We reviewed the postoperative course for details about postoperative outcomes such as severe postoperative intraabdominal sepsis (e.g., anastomotic leak, missed bowel injury, and rectal stump leak), urine culture results, Intensive care unit admission, stroke, myocardial infarction (MI), and mortality that occurred within the 30 days postoperative or until discharge. We defined CAUTI as the presence of a positive urine culture postoperatively in a patient with symptoms suggestive of urinary tract infection or sepsis. Our primary outcomes of interest were the incidence and the predictors of CAUTI among colorectal surgery patients. Our secondary outcome was the association of CAUTI with other adverse postoperative events.

Statistical methods

Descriptive statistics were used to calculate durations, means and standard deviation. Binominal logistic regression (univariate and multivariate regression) was used to test the predictors of the binary outcome variables. Shapiro-Wilk test was used to test the normality of the study samples. Simple frequency tables, cross-tabulations, and percentages were calculated. Linear Regression was used to test the predictors of the continuous outcome variable postoperative length of stay. Chi-square test was used to test and describe the relation between two categorized variables. Independent sample t-test analysis was applied to determine whether there was statistical evidence that the mean difference between two groups was significant or not significant. The level p < 0.05 was used as the cut-off value for significance. We performed statistical analysis using SPSS statistics for windows version 20.0 (IBM Corp., Armonk, NY, USA).

## Results

Out of 750 patients who underwent surgery on the colon and rectum, 130 had one or more exclusion criteria. We thus included 620 patients. In our study population, 55.3% were males, 29.2% were diabetic, 38.7% had urgent procedures, and 17.2% required at least one ureteric catheter to be placed. The full demographics, clinical and operative details of the included patients can be found in Table 1.

**Table 1:** Patient demographics and baseline laboratory investigations ASA: American Society of Anesthesiologists; WBC: white blood cells; PRBC: packed red blood cells

Table 1		n	Percent %	Mean ± SD
Age at operation (years)				53.99±16.9
GENDER	Male	343	55.3	
	Female	277	44.7	
BMI Kg/m^2^				26.18±6.23
smoking history	Non-smoker	333	82.8	
	Ex-smoker	20	5.0	
	Smoker	49	12.2	
Diabetes	Not diabetic	439	70.8	
	Diabetic	181	29.2	
Hypertension	No	417	67.3	
	Yes	203	32.7	
Ischemic heart disease	No	567	91.5	
	Yes	53	8.5	
Cerebrovascular disease	No	596	96.1	
	Yes	24	3.9	
Chronic kidney disease	No	574	92.6	
	Yes	46	7.4	
ASA classification	I	43	6.9	
	II	283	45.7	
	III	235	38.0	
	IV	55	8.9	
	V	3	.5	
Hemoglobin				11.29±2.36
WBC				8.13±5.45
Creatinine				90.73±81.44
Preop Albumen level				30.15±8
Preop International Normalized Ratio				1.1±0.21
Activated Partial Thromboplastin Time				32.69±10.02
Procedure Urgency	Routine	380	61.3	
	Emergency	240	38.7	
Regional Anesthesia	No	245	39.5	
	Yes	375	60.5	
Laparoscopic/Open Procedure	Laparoscopic	179	28.9	
	Open	441	71.1	
Rectal Surgery	No	487	78.5	
	Yes	133	21.5	
Preoperative Length of Stay (days)				5.66±11.36
Intraoperative PRBC Transfusion (units)	None	420	67.7	
	1	128	20.6	
	2	56	9.0	
	3	7	1.1	
	4	6	1.0	
	5	2	.3	
	6	1	.2	
Type of Urinary Catheter	Latex	30	5.1	
	Silicon	559	94.9	
Number of Ureteric Catheters	None	513	82.7	
	Single	33	5.3	
	Bilateral	74	11.9	
Regular Ureteric Catheter/Double-J	None	513	82.9	
	DJ	39	6.3	
	Catheter	67	10.8	

Out of 620 patients, 128 (20.6%) developed CAUTI. Factors that were associated with postoperative CAUTI in univariate analysis included older age, female gender, higher BMI, ASA class IV and V, lower hemoglobin, higher creatinine, lower albumin, higher WBC count, higher activated partial thromboplastin time (aPTT), urgent procedures, bilateral ureteric place placement, usage of double-J (DJ) stents, presence of postoperative abdominal sepsis, and perioperative steroid usage. Certain underlying medical conditions were also found to be associated with CAUTI, such as the presence of hypertension, ischemic heart disease, chronic kidney disease, cerebrovascular disease, and diabetes, Factors that were associated with CAUTI in multivariate analysis included lower hemoglobin, bilateral ureteric catheter placement, and postoperative abdominal sepsis. Full details of the univariate and multivariate analysis can be found Table 2.

**Table 2:** Univariate and multivariate analysis ASA: American Society of Anesthesiologists; INR: international normalized ratio; aPTT: activated partial thromboplastin time; PRBCs: packed red blood cells

Table 2		Univariant Analysis				Multivariate Analysis			
		OR	95% C.I.		p-value	OR	95% C.I.		p-value
			Lower	Upper			Lower	Upper	
Age		1.02	1.01	1.03	.001	1.01	0.99	1.03	.284
GENDER	Male	(Ref)							
	Female	1.66	1.12	2.46	.011	1.63	0.86	3.11	.135
BMI		1.03	1.00	1.06	.035	1.00	0.95	1.04	.845
Smoking History	Non-smoker	(Ref)							
	Ex-smoker	0.87	0.28	2.70	.816	0.86	0.23	3.14	.815
	Smoker	0.31	0.11	0.89	.030	0.36	0.11	1.18	.092
Hypertension	No	(Ref)							
	Yes	1.98	1.33	2.95	.001	1.54	0.75	3.18	.241
Ischemic Heart Disease	No	(Ref)							
	Yes	1.76	0.94	3.27	.076	1.30	0.46	3.67	.615
Chronic Kidney Disease	No	(Ref)							
	Yes	3.00	1.61	5.60	.001	2.09	0.61	7.19	.242
Diabetes	No	(Ref)							
	Yes	2.28	1.52	3.42	.000	1.06	0.54	2.09	.873
Cerebrovascular disease	No	(Ref)							
	Yes	2.89	1.25	6.68	.013	0.73	0.16	3.41	.692
ASA Classification	I	(Ref)							
	II	1.36	0.51	3.65	.540	2.31	0.27	19.90	.445
	III	2.49	0.94	6.63	.068	3.69	0.42	32.66	.240
	IV	4.34	1.47	12.82	.008	2.91	0.26	32.26	.384
	V	15.20	1.16	199.63	.038	3.11	0.05	177.36	.582
Preoperative Hemoglobin		0.89	0.82	0.97	.010	1.17	1.01	1.36	.037
Preoperative White blood cell count		1.04	1.01	1.07	.022	1.00	0.93	1.08	.967
Preoperative Albumin		0.95	0.93	0.97	.000	0.95	0.90	1.00	.057
Preoperative Creatinine		1.00	1.00	1.00	.026	1.00	1.00	1.01	.495
Preoperative INR		1.60	0.70	3.68	.263				
Preoperative aPTT		1.03	1.01	1.04	.006	1.03	0.99	1.06	.109
Preoperative Length of Stay		1.01	1.00	1.03	.114				
Procedure Urgency	Routine	(Ref)							
	Emergency	1.59	1.08	2.36	.020	0.89	0.41	1.96	.775
Regional Anasthesia	No	(Ref)							
	Yes	0.74	0.50	1.10	.133				
Rectal Surgery	No	(Ref)							
	Yes	1.44	0.92	2.25	.115				
Procedure Type	Laparoscopic	(Ref)							
	Open	1.50	0.95	2.36	.083	1.81	0.91	3.59	.090
U stent which side	None	(Ref)							
	Single	1.91	0.88	4.15	.101	1.28	0.36	4.55	.702
	Bilateral	1.98	1.16	3.41	.013	2.66	1.12	6.32	.026
Ureteric catheter used	None	(Ref)							
	Double J	3.06	1.56	6.02	.001	2.43	0.77	7.69	.132
	Regular Catheter	1.50	0.83	2.71	.184	2.43	0.77	7.69	.132
Urethral Catheter used	Latex								
	Silicon	0.85	0.36	2.03	.716				
Intraop PRBCs transfusion		1.17	0.95	1.45	.137				
Perioperative Steroid use	No	(Ref)							
	Yes	1.66	1.09	2.53	.019	1.33	0.66	2.66	.423
Postop Abdominal Sepsis	No	(Ref)							
	Yes	2.90	1.63	5.18	.000	2.95	1.23	7.07	.015

With regards to postoperative outcomes, we found that CAUTI was associated with increased risk of intensive care unit (ICU) stay, myocardial infarction (MI), stroke, and mortality in the first 30 days postoperatively. The details can be found in Table 3.

**Table 3:** Postoperative outcomes ICU: intensive care unit; MI: myocardial infarction

Table 3	p-value	Odd Ratio (OR)	95% CI for OR		
Postop complication					
			Lower	Upper	
Postop ICU stay	<0.001	2.675	1.793	3.993	
Postop MI	.001	2.411	1.414	4.112	
Postop stroke	.030	3.407	1.125	10.322	
Postop 30-day mortality	.006	2.445	1.298	4.607	

Using linear regression, we found that CAUTI was a significant predictor for increased postoperative length of stay, with the mean PLOS increased by 11 days for those who had CAUTI (the mean PLOS for those without CAUTI was 15.06 [SD 20.11], and for those who developed CAUTI 26.06 [SD 28.07]) (p < 0.001 and R = 0.20). Of note, postoperative length of stay was not normally distributed.

## Discussion

In this study, we found that 20.6% of patients undergoing colorectal surgery in our hospital population developed CAUTI. We found that CAUTI was associated with several adverse postoperative outcomes such as MI, stroke, PLOS, admission to the ICU, and mortality.

The risk of CAUTI was significantly elevated in our population compared to other studies that found it to be 4.1%-7.7% in colorectal surgery patients [1, 2, 6, 7]. We hypothesize that this maybe due to several factors. We included emergency procedures, while many series assessed only elective surgeries. Many studies have been performed at specialized centers where poorly educated and noncompliant patients maybe underrepresented. Diabetes, a significant predictor of CAUTI in our and other series, was highly prevalent in our patient population (29.2%), as about 19.6% of Saudis have it [8]. Our institution recently initiated an Enhanced Recovery After Surgery (ERAS) protocol, and most of the patients in our study preceded this protocol. The ERAS protocols promote earlier removal of urinary catheters. Though we did not assess duration of catheterization in our study population, it has been shown previously that CAUTI increases by 5-10% per catheter-day after the first 48 hours of catheterization [9].

We noted several associations between CAUTI and patient characteristics, lab values, and operative details. Some factors such as patient age, gender, ASA score, steroid use, diabetes, hypertension, and a history of cerebrovascular disease have been previously identified as risk factors for CAUTI after colorectal surgery [1,2,4]. Others such as preoperative hemoglobin, albumin, WBC count, hypertension, CKD, and emergency surgery have been identified as risk factors for morbidity and mortality after colorectal surgery in general [7,10,11]. Some of these factors, such as WBC and aPTT, are more likely to be abnormal in an emergency situation, for which prolonged catheterized maybe necessary. We also found it to be associated with postoperative abdominal sepsis (which we defined as anastomotic leak, missed enterotomy, and rectal stump leakage). We believe it is probably related to the need for prolonged catheterizations due to the development of these significant postoperative complication. We found a history of smoking to be inversely related to the risk of CAUTI. This maybe due to underreporting of smoking history, which could affect the validity of this result.

We found no association between CAUTI and IHD, BMI, regional anesthesia, type of surgery (laparoscopic vs open), and rectal surgery. This is despite other studies having found an association with some of these factors [1,4].

We found that the type of Foley catheter was not associated with CAUTI. This is consistent with previous studies. A Cochrane review found no difference between CAUTI risk among standard Foley catheters. It did note a reduction in the risk of UTI and bacteriuria with silver alloy and antibiotic-impregnated Foley catheters, respectively [12]. We found that the presence, number, and type of ureteric stent was associated with the risk of CAUTI. In our institution, when a surgeon requests preoperative placement of a ureteric catheter, the type and size of ureteric catheter placed is left to the discretion of the urologist. It has been documented that DJ stents are associated with an increased risk of bacteriruria [13]. Additionally, vesicoureteric reflux has been noted in patients with DJ stents [14]. This combination could potentially lead to increased risk of acute pyelonephritis and sepsis [15] in patients who develop CAUTI, as when placed preoperatively in the setting of colorectal surgery, they will often remain in place until they can be removed electively later. We did not assess if patients with CAUTI and DJ stents had a worse outcome than those without DJ stents, and this maybe an avenue for further research. If this were found to be true, then it would suggest that simple ureteric catheters should be chosen over DJ stents.

We found that CAUTI was associated with increased risk of ICU admission, MI, stroke, increased length of stay, and 30-day mortality [4]. This is similar to the increased risk of overall morbidity associated with CAUTI [4, 16]. This may be multifactorial. Indwelling catheters limit mobility, potentially leading to an increased incidence of venous thromboembolism (VTE) [5]. Other research has shown that CAUTI is associated with the development of systemic sepsis in 3.6% of patients [9]. It may also occur that CAUTI can develop secondary to the requirement for prolonged catheterization from another postoperative morbidity, such as a stroke or unplanned reintubation.

An additional concern with the development of CAUTI in the postoperative period is that it can have long-term effects. Previous research that assessed the effect of postoperative CAUTI on survival after major surgery (of any type) found that the occurrence of a UTI was associated with a mortality rate of 6% at 30 days, 25% at one year, and 58% at five years [17]. It has been shown that infectious postoperative complications in colorectal cancer patients is associated with decreased long-term survival [18]. Potential reasons could be that infectious complications affect cell mediated immunity, leading to increased risk of cancer recurrence, or those who develop significant complications are less likely to receive adjuvant therapy [18].

Many methods, some of them at minimal cost, have been found to reduce the risk of CAUTI. These include daily electronic prompts to remove the Foley catheter, implementation of an ERAS protocol, usage of closed catheter drainage systems, minimizing disconnection of the catheter junction, placement of the urine bag below the level of the bladder, and consideration of usage of antimicrobial-coated urinary catheters, among others. Prophylactic antibiotics for CAUTI are not recommended [16, 19, 20]. Some authors have suggested routine suprapubic catheters in abdominal surgery instead of Foley catheters to decrease the risk of CAUTI, but an randomized controlled trial (RCT) showed no difference in risk of CAUTI [21].

Limitations

Our study was a retrospective study. Our study sample is on the smaller side compared to other studies that have assessed postoperative CAUTI. We did not assess the duration of catheterization, an important factor that predicts the occurrence of CAUTI, as this information was not readily available to us. We did not assess the association of CAUTI with prolonged operative time. Though we have shown that CAUTI is associated with other postoperative complications, it is difficult to determine if it is a risk factor for them or the other adverse events lead to a longer catheterization time and thus an increased risk of CAUTI.

## Conclusions

Catheter-associated urinary tract infection is a significant cause of morbidity after colorectal surgery. Our patient population had a significantly higher risk of CAUTI compared to other series. Attempts to identify the underlying reasons for this higher CAUTI risk and steps to reduce it at an institutional level are essential. Though sometimes labelled a minor postoperative complication, the occurrence of CAUTI is associated with other more significant postoperative complications, including death. A new finding in our study was the association between the risk of CAUTI with bilateral ureteric catheterization and DJ stents in colorectal surgery. Future research should look into the superiority of one method of ureteric instrumentation over another in reducing the risk of urinary tract infection in a randomized setting.

## References

[1] Regenbogen SE, Read TE, Roberts PL, Marcello PW, Schoetz DJ, Ricciardi R (2011). Urinary tract infection after colon and rectal resections: more common than predicted by risk-adjustment models. J Am Coll Surg.

[2] Attaluri V, Kiran RP, Vogel J, Remzi F, Church J (2011). Risk factors for urinary tract infections in colorectal compared with vascular surgery: a need to review current present-on-admission policy?. J Am Coll Surg.

[3] Dimick JB, Chen SL, Taheri PA, Henderson WG, Khuri SF, Campbell DA Jr (2004). Hospital costs associated with surgical complications: a report from the private-sector National Surgical Quality Improvement Program. J Am Coll Surg.

[4] Sheka AC, Tevis S, Kennedy GD (2016). Urinary tract infection after surgery for colorectal malignancy: risk factors and complications. Am J Surg.

[5] Holroyd-Leduc JM, Sen S, Bertenthal D (2007). The relationship of indwelling urinary catheters to death, length of hospital stay, functional decline, and nursing home admission in hospitalized older medical patients. J Am Geriatr Soc.

[6] Kin C, Rhoads KF, Jalali M, Shelton AA, Welton ML (2013). Predictors of postoperative urinary retention after colorectal surgery. Dis Colon Rectum.

[7] Alves A, Panis Y, Mathieu P, Mantion G, Kwiatkowski F, Slim K (2005). Postoperative mortality and morbidity in French patients undergoing colorectal surgery: results of a prospective multicenter study. Arch Surg.

[8] Whiting DR, Guariguata L, Weil C, Shaw J (2011). IDF diabetes atlas: global estimates of the prevalence of diabetes for 2011 and 2030. Diabetes Res Clin Pract.

[9] Wald HL, Ma A, Bratzler DW, Kramer AM (2008). Indwelling urinary catheter use in the postoperative period: analysis of the national surgical infection prevention project data. Arch Surg.

[10] Longo WE, Virgo KS, Johnson FE (2000). Risk factors for morbidity and mortality after colectomy for colon cancer. Dis Colon Rectum.

[11] Masoomi H, Kang CY, Chen A, Mills S, Dolich MO, Carmichael JC, Stamos MJ (2012). Predictive factors of in-hospital mortality in colon and rectal surgery. J Am Coll Surg.

[12] Schumm K, Lam TB (2008). Types of urethral catheters for management of short-term voiding problems in hospitalised adults. Cochrane Database Syst Rev.

[13] Farsi HM, Mosli HA, Al-Zemaity MF, Bahnassy AA, Alvarez M (1995). Bacteriuria and colonization of double-pigtail ureteral stents: long-term experience with 237 patients. J Endourol.

[14] Mosli HA, Farsi HM, al-Zimaity MF, Saleh TR, al-Zamzami MM (1991). Vesicoureteral reflux in patients with double pigtail stents. J Urol.

[15] Chen SJ, Huang CP, Chiu KY (2021). Association of acute pyelonephritis with double-J ureteral stenting: a nationwide population-based case control study. Scand J Urol.

[16] Nagle D, Curran T, Anez-Bustillos L, Poylin V (2014). Reducing urinary tract infections in colon and rectal surgery. Dis Colon Rectum.

[17] Khuri SF, Henderson WG, DePalma RG, Mosca C, Healey NA, Kumbhani DJ (2005). Determinants of long-term survival after major surgery and the adverse effect of postoperative complications. Ann Surg.

[18] Artinyan A, Orcutt ST, Anaya DA, Richardson P, Chen GJ, Berger DH (2015). Infectious postoperative complications decrease long-term survival in patients undergoing curative surgery for colorectal cancer: a study of 12,075 patients. Ann Surg.

[19] Hooton TM, Bradley SF, Cardenas DD (2010). Diagnosis, prevention, and treatment of catheter-associated urinary tract infection in adults: 2009 International Clinical Practice Guidelines from the Infectious Diseases Society of America. Clin Infect Dis.

[20] Miller TE, Thacker JK, White WD (2014). Reduced length of hospital stay in colorectal surgery after implementation of an enhanced recovery protocol. Anesth Analg.

[21] Baan AH, Vermeulen H, van der Meulen J, Bossuyt P, Olszyna D, Gouma DJ (2003). The effect of suprapubic catheterization versus transurethral catheterization after abdominal surgery on urinary tract infection: a randomized controlled trial. Dig Surg.

